# Retrospective analysis of independent predictors of progression‐free survival in patients with *EGFR* mutation‐positive advanced non‐small cell lung cancer receiving first‐line osimertinib

**DOI:** 10.1111/1759-7714.14608

**Published:** 2022-08-18

**Authors:** Shuhei Teranishi, Chihiro Sugimoto, Satoshi Nagaoka, Hirokazu Nagayama, Wataru Segawa, Atsushi Miyasaka, Shuntaro Hiro, Yukihito Kajita, Chihiro Maeda, Nobuaki Kobayashi, Masaki Yamamoto, Makoto Kudo, Takeshi Kaneko

**Affiliations:** ^1^ Respiratory Disease Center Yokohama City University Medical Center Yokohama Japan; ^2^ Department of Pulmonology Yokohama City University Graduate School of Medicine Yokohama Japan

**Keywords:** EGFR, non‐small cell lung cancer, osimertinib, PD‐L1 TPS, performance status

## Abstract

**Background:**

Clinically measurable factors affecting the progression‐free survival (PFS) of patients receiving osimertinib as first‐line therapy for epidermal growth factor receptor (EGFR) mutation‐positive advanced non‐small cell lung cancer (NSCLC) have not yet been established.

**Methods:**

We retrospectively reviewed the medical records of 61 patients treated with osimertinib as primary therapy for *EGFR* mutation‐positive advanced NSCLC at Yokohama City University Medical Center between August 2018 and March 2022. Our objective was to identify the independent predictors of PFS.

**Results:**

The median age of participants was 74 years. Overall, 73.8% had good (0–1) Eastern Cooperative Oncology Group performance status (PS), and 98.4% had histology of adenocarcinoma. The *EGFR* mutation was exon19 deletion in 52.5% and exon21 L858R in 44.3% of patients. Programmed death‐ligand 1 tumor proportion score >50% was observed in 21.3% and liver metastasis in 9.9% of patients. Median PFS was 19.5 months (95% confidence interval [CI]: 10.6–31.6), and overall survival was not reached. The objective response rate was 68.9%, and disease control rate was 93.4%. Multivariate analysis showed that poor PS (2–4) negatively impacted PFS (hazard ratio, 3.79; 95% CI: 1.46–9.87; *p* = 0.006). Median PFS in the good PS and poor PS groups was 20.4 months (95% CI: 12.4‐not evaluable) and 7.2 months (95% CI: 7.2–19.5), respectively. Interstitial lung disease of all grades and grade 3 was observed as an adverse event in 6.6 and 4.9% of patients, respectively.

**Conclusion:**

Poor PS was associated with poor prognosis in patients with *EGFR* mutation‐positive advanced NSCLC treated with osimertinib as first‐line therapy.

## Graphical abstract

We aimed to identify, among clinically measurable factors, independent predictors of progression‐free survival of patients who received osimertinib as first‐line treatment for EGFR‐mutant advanced non‐small cell lung cancer. As a result, poor PS (2–4) was an independent predictor with a negative impact on progression‐free survival (HR = 3.79, 95% CI: 1.46–9.87, *p* = 0.006).

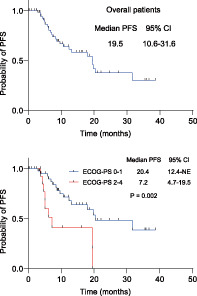

## INTRODUCTION

Recently, gene analysis technology has led to personalized treatment for non‐small cell lung cancer (NSCLC). In Caucasian and Asian NSCLC patients, epidermal growth factor receptor (EGFR) mutations account for approximately 10% and 40%–55% of cases.^1^, ^2^, ^3^ In EGFR mutation‐positive (EGFRm+) advanced NSCLC patients, first/second‐generation EGFR tyrosine kinase inhibitors (TKIs) have been demonstrated to prolong progression‐free survival (PFS) compared to cytotoxic agents.^4^, ^5^, ^6^ The phase III AURA trial reported that osimertinib, a third‐generation EGFR‐TKI, prolonged PFS compared to cytotoxic agents in patients with T790M mutation‐mediated resistance after treatment with first/second‐generation EGFR‐TKIs.^7^ The phase III FLAURA trial reported that osimertinib prolonged PFS and overall survival (OS) compared to first‐generation EGFR‐TKIs in the first‐line treatment of patients with EGFRm+ advanced NSCLC.^8^, ^9^ However, when these EGFR‐TKIs are used as first‐line therapy, approximately 20%–30% of patients do not respond at all or only respond for a brief period (<3 months).^10^ Therefore, researchers are attempting to identify factors that predict response to therapy.

As existing phase III trials for third‐generation EGFR‐TKIs exclude poor PS cases and patients with specific comorbidities, their results are not directly applicable to the real world. There is, however, ample real‐world research on first/second‐generation EGFR‐TKI use in the first‐line treatment of patients with EGFRm+ advanced NSCLC, and these reports have identified factors negatively affecting PFS.^11^, ^12^, ^13^ On weighing these factors, poor PS and liver metastasis emerged as important predictors.^14^ Recently, high expression of programmed death‐ligand 1 (PD‐L1) tumor proportion score (TPS), which predicts favorable treatment response in immune checkpoint inhibitor (ICI), has also been reported to negatively impact PFS in first‐line therapy with EGFR‐TKIs.^15^, ^16^, ^17^, ^18^

Real‐world data on the use of osimertinib as first‐line treatment for patients with EGFRm+ advanced NSCLC are scarce and consist primarily of the Phase III FLAURA trial and its subset.^8^, ^19^, ^20^ However, the FLAURA trial excluded patients with poor PS, and although it reported that PD‐L1 TPS did not affect PFS, only 1% PD‐L1 TPS was used as the cutoff value. In the limited real‐world evidence available, poor PS is not reported as an independent predictor of PFS,^21^, ^22^ and patients with PD‐L1 TPS > 50% are shown to have shorter PFS and OS.^23^ However, further validation is needed to confirm these findings.

In this single‐center retrospective study, we aimed to identify, among clinically measurable factors, independent predictors of PFS of patients who received osimertinib as first‐line treatment for EGFRm+ advanced NSCLC.

## METHODS

### Study design and patients

This was a single‐center, retrospective study conducted at Yokohama City University Medical Center, Japan. The study was conducted by the provisions of the Declaration of Helsinki and was approved by the Ethics Committee of Yokohama City University (approval number: F220400009).

The study included patients with EGFRm+ advanced NSCLC who received osimertinib as first‐line therapy from August 2018 to March 2022. Eligibility criteria included the following: (a) NSCLC with *EGFR* mutations detected in tissue or cell samples between January 2017 and March 2022; (b) advanced stage of disease or postoperative relapse; and (c) first‐line therapy with osimertinib. Patients with positive EGFR exon 20 insertion mutation and/or first‐line treatment with any drug other than osimertinib were excluded. Data on each patient's age, sex, smoking status, comorbidity, Eastern Cooperative Oncology Group performance status (ECOG‐PS), histology, disease stage, brain metastases, liver metastases, *EGFR* mutation type, and PD‐L1 expression status were collected. We also recorded the tumor response to osimertinib and patients' PFS and OS. The cutoff date was March 10, 2022.

We defined an ECOG‐PS of 0–1 as good and 2–4 as poor. Comorbidity was scored using the Charlson comorbidity index (CCI). A cutoff value of 2 or 3 points is recommended for diseases with high mortality rates, and this study set the cutoff at 2 points.^24^ With the aging of the population worldwide, approximately 37% of newly diagnosed lung cancer patients are over 75 years of age,^25^ but the subset analysis of the FLAURA trial only set the cutoff age at 65 years.^8^ In addition, a trial evaluating the efficacy of first‐line osimertinib in patients with EGFRm+ advanced NSCLC aged 75 years or older showed favorable PFS.^26^ For these reasons, we used 75 years as the age cutoff.

### Treatment response and adverse event evaluation

EGFRm+ advanced NSCLC patients were treated with osimertinib until the disease progressed or an unacceptable adverse event occurred. We used computed tomography (CT) to determine treatment response. CT was performed every 3 months or whenever the patient's general condition worsened, or an adverse event occurred. The tumor response was determined using the Response Evaluation Criteria in Solid Tumors (RECIST) version 1.1^27^ as follows: complete response (CR) indicated complete disappearance of the tumor; partial response (PR), reduction in the sum of tumor diameters by 30% or more; stable disease (SD), no change in tumor size; and progressive disease (PD), a significant increase in the sum of tumor diameters by 20% or more and an absolute increase of ≥5 mm or the appearance of a new lesion. We did not investigate resistance mechanisms when it came to PD against osimertinib. Objective response was defined as CR or PR, and disease control as CR, PR, or SD. PFS was defined as the time from the first day of osimertinib treatment to disease progression or death. Patients who were alive and did not show disease progression by the cutoff date were considered censored. OS was defined as the time from the first day of osimertinib treatment to death. Patients who had not died by the cutoff date were considered censored. Adverse events were assessed using the National Cancer Institute Common Terminology Criteria for Adverse Events, version 5.0.

### Testing for *EGFR* mutations and PD‐L1 expression

*EGFR* gene mutations were detected in tumor samples before osimertinib initiation using the Cobas EGFR Mutation Test (Roche Molecular Systems Inc.) or the Oncomine Dx Target Test Multi CDx (Thermo Fisher Scientific Inc.). Immunohistochemical analysis of PD‐L1 expression was performed by an experienced pathologist using PD‐L1 22C3 pharmDx (Dako) on tumor samples before osimertinib initiation. At least 100 tumor cells were counted, and the percentage of tumor cells expressing PD‐L1 was determined and designated as TPS.

### Statistical analysis

PFS and OS were plotted by the Kaplan–Meier method, and the log‐rank test was used for comparison. For PFS, univariate Cox proportional hazards analysis was performed for age, sex, smoking status, CCI, PS, stage, brain metastases, liver metastases, EGFR genotype, and PD‐L1 TPS. Each factor's hazard ratio (HR) was calculated with 95% confidence interval (CI). Multivariate Cox proportional hazards analysis was performed to identify independent predictors of PFS by including factors, such as PS, liver metastasis, and PD‐L1 TPS, which are reported as independent factors associated with PFS in previous studies, and factors for which *p* was <0.10 in univariate analysis. We used the chi‐square test or Fisher's exact test to compare the proportions of categorical variables between the groups. Significance was determined by a two‐tailed test with *p* < 0.05. Statistical analyses were performed using GraphPad Prism 9 (GraphPad Software).

## RESULTS

### Patient characteristics

**FIGURE 1:**
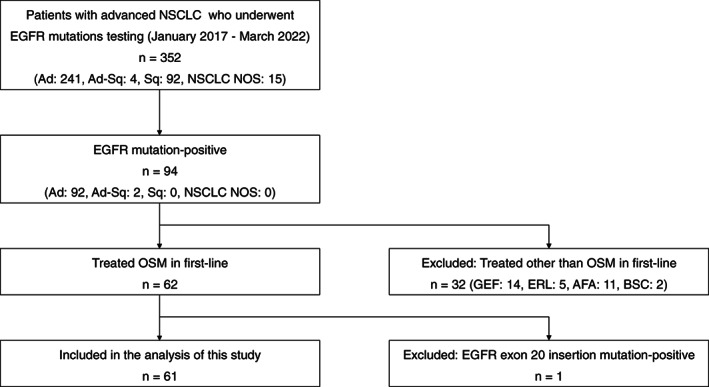
Flow diagram for patient selection. Ad, adenocarcinoma; Ad‐Sq, adeno‐squamous cell carcinoma; AFA, afatinib; BSC, best supportive care; EGFR, epidermal growth factor receptor; ERL, erlotinib; GEF, gefitinib; NOS, not otherwise specified; NSCLC, non‐small cell lung cancer; OSM, Osimertinib; Sq, squamous cell carcinoma

Figure 1 shows the patient selection flowchart. Of the 352 patients with advanced NSCLC who underwent Cobas EGFR Mutation Test or Oncomine Dx Target Test Multi CDx between January 2017 and March 2022, *EGFR* mutations were detected in 94 patients. Of these, 62 patients received osimertinib as first‐line therapy. A total of 61 patients were included in the study one patient with a positive *EGFR* exon 20 insertion mutation was excluded. Since August 2018, first‐line therapy with osimertinib is covered under insurance for patients with EGFRm+ advanced NSCLC in Japan. All 61 patients started primary chemotherapy with osimertinib after August 2018.

Table 1 shows the patient characteristics. The median age was 74 years, 37 (60.7%) were female, and eight (13.1%) had a CCI score of 2 or higher. Table 2 shows detailed data on comorbidities at baseline. The PS was good in 45 patients (73.8%) and poor in 16 patients (26.2%), with no patient having a PS of 4. The histological type was adeno‐squamous carcinoma in only one case (1.6%) and adenocarcinoma in all others. Exon 19 deletions were found in 32 cases (52.5%) and exon 21 L858R point mutation in 27 cases (44.3%). Seventeen patients (27.9%) had brain metastases (solid), and there were no cases of leptomeningeal carcinomatosis. Six of the 17 patients with brain metastases were symptomatic, five received stereotactic radiotherapy, and one received whole brain irradiation. Of the 11 asymptomatic patients, two with brain stem metastases received stereotactic radiotherapy, while the remaining nine received osimertinib for brain metastases. Six patients (9.9%) had liver metastases. PD‐L1 TPS was <1% in 30 patients (49.2%), 1–49% in 18 patients (29.5%), and > 50% in 13 patients (21.3%).

**TABLE 1:** Patient characteristics

Characteristics	n (%), *n* = 61
Age (years)	
Median (IQR)	74 (64–78)
<75	34 (55.7)
≥75	27 (44.3)
Sex	
Female	37 (60.7)
Male	24 (39.3)
Smoking status	
Current or former	28 (45.9)
Never	33 (54.1)
CCI	
<2	53 (86.9)
≥2	8 (13.1)
ECOG‐PS	
0–1	45 (73.8)
2–4	16 (26.2)
Histology	
Adenocarcinoma	60 (98.4)
Adeno‐squamous cell carcinoma	1 (1.6)
EGFR mutation	
19 del	32 (52.5)
L858R	27 (44.3)
L861Q	2 (3.2)
stage	
III	2 (3.3)
IV	38 (62.3)
Postoperative recurrence	21 (34.4)
Brain metastasis (solid)	
Negative	44 (72.1)
Positive	17 (27.9)
Liver metastasis	
Negative	55 (90.1)
Positive	6 (9.9)
PD‐L1 TPS	
<1%	30 (49.2)
1–49%	18 (29.5)
≥50%	13 (21.3)

Abbreviations: 19 del, exon 19 deletion; CCI, Charlson comorbidity index; ECOG‐PS, Eastern Cooperative Oncology Group performance status; EGFR, epidermal growth factor receptor; IQR, interquartile range; L858R, exon 21 L858R mutation; L861Q, exon 21 L861Q mutation; PD‐L1 TPS, programmed cell death‐ligand 1 tumor proportion score.

**TABLE 2:** Comorbidities based on the Charlson comorbidity index

	n (%), *n* = 61
Diabetes^a^	10 (16.4)
Cancer without metastases^b^	4 (6.6)
Congestive heart failure^c^	4 (6.6)
Connective tissue disease	2 (3.3)
Moderate or severe renal disease^d^	2 (3.3)
Myocardical infarction	2 (3.3)
Ulcer disease	2 (3.3)
Cerebrovascular disease	1 (1.6)
Chronic pulmonary disease^e^	1 (1.6)
Mild liver disease^f^	1 (1.6)

^a^ Currently treated with oral diabetes medications or insulin.

^b^ Solid cancer with no metastases and a history of treatment within 5 years.

^c^ Exertional or paroxysmal nocturnal dyspnea that has responded to pharmacological therapy.

^d^ Serum creatinine >3.0 mg/dl, post maintenance hemodialysis, renal transplantation, coexisting uremia.

^e^ Leading to dyspnea even with mild exertion.

^f^ Chronic hepatitis or cirrhosis without portal hypertension.

### Clinical efficacy of osimertinib

**FIGURE 2:**
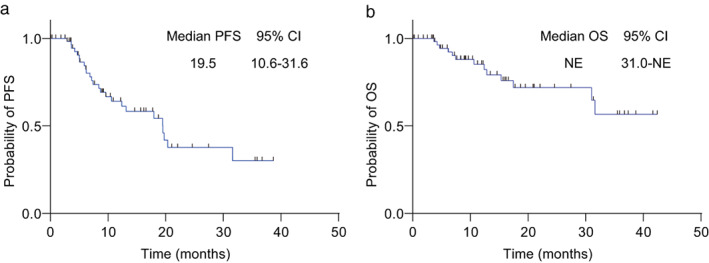
The efficacy of osimertinib in *EGFR*‐mutant advanced NSCLC patients. (a) Kaplan–Meier survival curves of PFS for all patients. (b) Kaplan–Meier survival curves of OS for all patients. CI, confidence interval; EGFR, epidermal growth factor receptor; NE, not evaluable; NSCLC, non‐small cell lung cancer; OS, overall survival; PFS, progression‐free survival

Median PFS was 19.5 months (95% CI: 10.6–31.6) (Figure 2a), and median OS was not reached (Figure 2b). Tumor response was CR in three patients (4.9%), PR in 39 patients (63.9%), and SD in 15 patients (24.6%). The objective response was 42 (68.9%), and disease control was achieved in 57 (93.4%) patients (Table 3).

**TABLE 3:** Treatment response

n (%), *n* = 61		
Complete response	3 (4.9)	
Partial response	39 (63.9)	
Stable disease	15 (24.6)	
Progressive disease	0	
Not evaluable	4 (6.6)	
	Yes	No
Objective response	42 (68.9)	19 (31.1)
Disease control	57 (93.4)	4 (6.6)

### Univariate and multivariate analysis of factors associated with PFS

Table 4 shows the results of univariate and multivariate analyses of factors associated with PFS after first‐line osimertinib treatment. Univariate Cox proportional hazards analysis revealed that age 75 years or older (HR = 2.27, 95% CI: 1.01–5.09, *p* = 0.047) and poor PS (HR = 3.54, 95% CI: 1.49–8.39, *p* = 0.004) were significantly associated with shorter PFS. We then performed a multivariate analysis including these two factors (age, PS) and data on liver metastasis and PD‐L1 TPS, which have been previously reported as independent predictors of PFS. The results revealed that only poor PS was significantly associated with shorter PFS (HR = 3.79, 95% CI: 1.46–9.87, *p* = 0.006).

**TABLE 4:** Univariate and multivariate analysis for factors affecting PFS

	Univariate analysis		Multivariate analysis	
Category	Hazard ratio (95% CI)	*p*‐value	Hazard ratio (95% CI)	*p*‐value
Age (years)				
<75	1		1	
≥75	2.27 (1.01–5.09)	0.047	2.31 (0.96–5.53)	0.069
Sex				
Female	1			
Male	1.20 (0.53–2.68)	0.662		
Smoking status				
Current or former	1			
Never	1.26 (0.56–2.87)	0.577		
CCI				
<2	1			
≥2	1.79 (0.67–4.82)	0.247		
ECOG PS				
0–1	1		1	
2–4	3.54 (1.49–8.39)	0.004	3.79 (1.46–9.87)	0.006
*EGFR* mutation				
19 del	1			
L858R	1.76 (0.78–3.98)	0.172		
L861Q	1.98 (0.25–15.86)	0.517		
stage				
III	1.39 (0.18–10.82)	0.752		
IV	1			
Postoperative recurrence	1.16 (0.52–2.62)	0.709		
Brain metastasis (solid)				
Negative	1			
Positive	1.96 (0.85–4.48)	0.113		
Liver metastasis				
Negative	1		1	
Positive	0.53 (0.12–2.28)	0.393	0.89 (0.19–4.12)	0.886
PD‐L1 TPS				
<50%	1		1	
≥50%	1.44 (0.58–3.63)	0.433	0.63 (0.22–1.81)	0.392

Abbreviations: 19 del, exon 19 deletion; CCI, Charlson comorbidity index; CI, confidential interval; ECOG PS, Eastern Cooperative Oncology Group performance status; EGFR, epidermal growth factor receptor; L858R, exon 21 L858R mutation; L861Q, exon 21 L861Q mutation; PD‐L1 TPS, programmed cell death‐ligand 1 tumor proportion score; PFS, progression‐free survival.

#### PFS by subgroup

**FIGURE 3:**
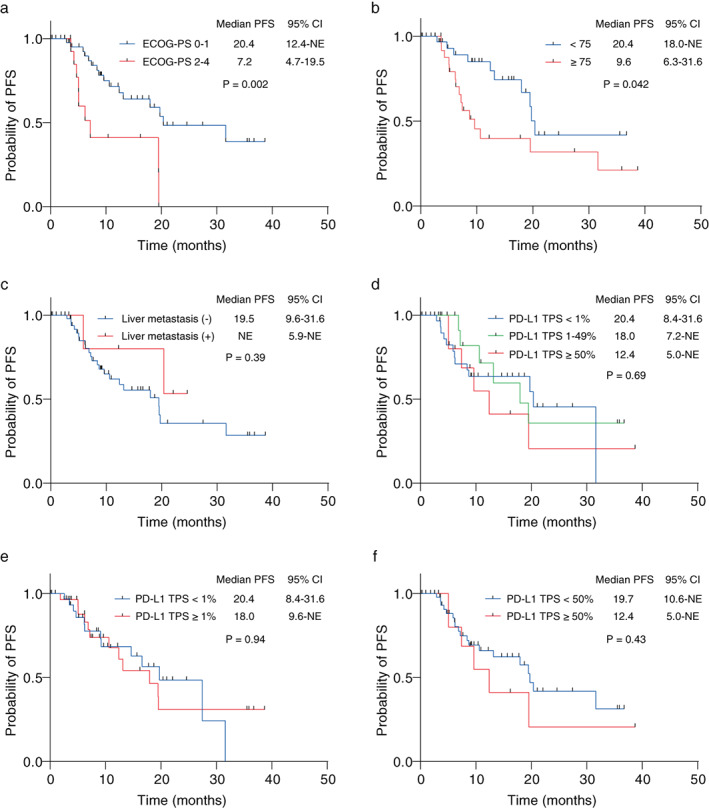
The efficacy of osimertinib in *EGFR*‐mutant advanced NSCLC patients by subgroup. (a) Kaplan–Meier survival curves of PFS by patients ECOG‐PS 0–1 and 2–4. (b) Kaplan–Meier survival curves of PFS by patients <75 years and ≥75 years. (c) Kaplan–Meier survival curves of PFS by patients with and without liver metastases. (d, e, f) Kaplan–Meier survival curves of PFS by patients according to PD‐L1 TPS. CI, confidence interval; ECOG‐PS, Eastern Cooperative Oncology Group performance status; EGFR, epidermal growth factor receptor; NE, not evaluable; NSCLC, non‐small cell lung cancer;PD‐L1 TPS, programmed cell death‐ligand 1 tumor proportion score; PFS, progression‐free survival

Figure 3 shows PFS by subgroup. The median PFS was 20.4 months for patients with a good PS (95% CI: 12.4–not evaluable [NE]) and 7.2 months for those with a poor PS (95% CI: 4.7–19.5) (Figure 3a). The median PFS was 20.4 months (95% CI: 18.0‐NE) for patients <75 years old and 9.6 months (95% CI: 6.3–31.6) for patients >75 years old (Figure 3b). Additionally, the median PFS was 19.5 months (95% CI: 9.6–31.6) in the group without liver metastases and was not reached in the group with liver metastases (Figure 3c). For PD‐L1 TPS, we divided the data into three categories. The median PFS was 20.4 months (95% CI: 8.4–31.6) for TPS <1%, 18.0 months (95% CI: 7.2‐NE) for TPS from 1–49%, and 12.4 months (5.0‐NE) for TPS ≥50% (Figure 3d). For TPS ≥1%, the duration to PFS was 18.0 months (9.6‐NE) (Figure 3e), and for <50%, it was 19.7 months (95% CI: 10.6–NE) (Figure 3f).

### Adverse events during osimertinib treatment

**FIGURE 4:**
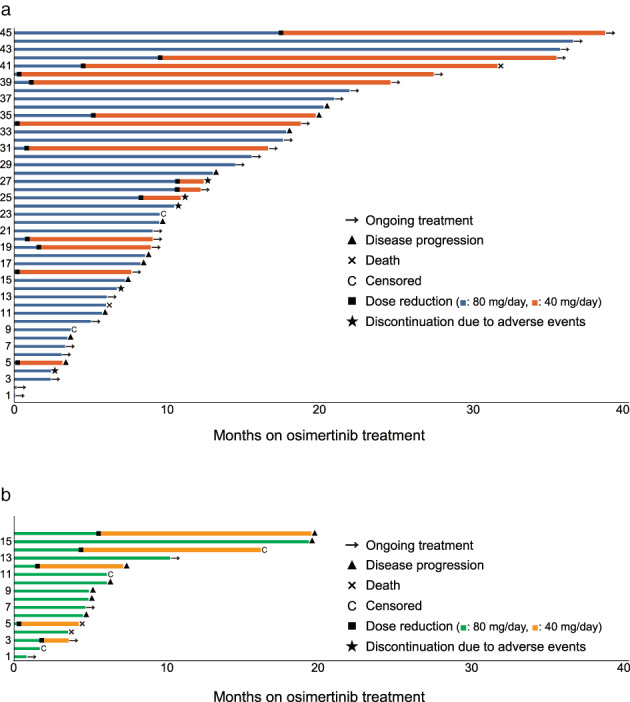
Detailed individual progress on first‐line osimertinib in *EGFR*‐mutant advanced NSCLC patients. (a) ECOG‐PS good (0–1) patients. (b) ECOG‐PS poor (2–4) patients. ECOG‐PS, Eastern Cooperative Oncology Group performance status; EGFR, epidermal growth factor receptor; NSCLC, non‐small cell lung cancer

Table 5 shows the adverse events observed during treatment with osimertinib. The most common adverse event was skin rash of any grade in 26 patients (42.6%) and grade 3 or higher in two patients (3.3%). Diarrhea (15 [24.6%] of any grade, no grade 3 or higher) and paronychia (14 [23.0%] of any grade, no grade 3 or higher) were next in frequency. Interstitial lung disease of any grade was reported in four patients (6.6%) and of grade 3 or higher in three patients (4.9%). Adverse events requiring osimertinib dose reduction were observed in 20 patients (32.8%). Five patients (8.2%) had adverse events requiring discontinuation of osimertinib; causes for discontinuation were interstitial lung disease in three patients (4.9%), thrombocytopenia in one patient (1.6%), and elevated creatinine in one patient (1.6%). The detailed treatment course of each patient is shown in Figure 4a,b, dividing them into good PS and poor PS groups.

**TABLE 5:** Adverse events during osimertinib treatment

Adverse events, n (%), *n* = 61						
n (%), *n* = 61	Grade 1	Grade 2	Grade 3	Grade 4, 5	All grades	Grade ≥3
Rash	16 (26.2)	8 (13.1)	2 (3.3)	0	26 (42.6)	2 (3.3)
Diarrhea	14 (23.0)	1 (1.6)	0	0	15 (24.6)	0
Paronychia	10 (16.4)	4 (6.6)	0	0	14 (23.0)	0
ALT increased	5 (8.2)	0	2 (3.3)	0	7 (11.5)	2 (3.3)
AST increased	3 (4.9)	0	2 (3.3)	0	5 (8.2)	2 (3.3)
Neutropenia	1 (1.6)	2 (3.3)	2 (3.3)	0	5 (8.2)	2 (3.3)
Anorexia	0	4 (6.6)	0	0	4 (6.6)	0
Interstitial lung disease	1 (1.6)	0	3 (4.9)	0	4 (6.6)	3 (4.9)
Oral mucositis	2 (3.3)	1 (1.6)	0	0	3 (4.9)	0
Thrombocytopenia	1 (1.6)	0	2 (3.3)	0	3 (4.9)	2 (3.3)
Creatinine increased	1 (1.6)	1 (1.6)	0	0	2 (3.3)	0
Creatine kinase increased	2 (3.3)	0	0	0	2 (3.3)	0
Fatigue	0	1 (1.6)	1 (1.6)	0	2 (3.3)	1 (1.6)
Nausea	0	1 (1.6)	1 (1.6)	0	2 (3.3)	1 (1.6)
QTc prolongation	1 (1.6)	0	1 (1.6)	0	2 (3.3)	1 (1.6)
Anemia	0	0	1 (1.6)	0	1 (1.6)	1 (1.6)
Constipation	1 (1.6)	0	0	0	1 (1.6)	0
Dizziness	1 (1.6)	0	0	0	1 (1.6)	0
Dysgeusia	1 (1.6)	0	0	0	1 (1.6)	0
Dyspnea	1 (1.6)	0	0	0	1 (1.6)	0

Abbreviations: ALT, alanine transaminase; AST, aspartate transaminase.

### Treatment after disease progression

Of the 18 patients who discontinued first‐line osimertinib due to disease progression, 11 (61.1%) went on to receive second‐line therapy. The regimens used were platinum combination therapy in six patients, platinum combination therapy plus ICI in three patients, ICI alone in one patient, and other EGFR‐TKI in one patient.

## DISCUSSION

Our study demonstrates the efficacy of first‐line osimertinib in real‐world patients with EGFRm+ advanced NSCLC. PS affected the clinical outcome of first‐line osimertinib. Patients with poor PS had shorter PFS than those with good PS.

In the FLAURA trial, a phase III study of first‐line osimertinib therapy in EGFRm+ advanced NSCLC patients, the median PFS was 18.9 months (95% CI: 15.2–21.4),^8^ and median OS was 38.6 months (95% CI: 34.5–41.8).^9^ In this study, the median PFS was 19.5 months (95% CI: 10.6–31.6), and the median OS was not reached. These results are consistent with those of the FLAURA study and demonstrate the real‐world efficacy of first‐line osimertinib therapy for EGFRm+ advanced NSCLC.

The median PFS in this study was 7.2 months (95% CI: 4.7–19.5) for patients with poor PS and 20.4 months (95% CI: 12.4‐NE) for those with good PS. The difference in PFS was statistically significant, and poor PS was an independent predictor with a negative impact on PFS (HR = 3.79, 95% CI: 1.46–9.87, *p* = 0.006). In studies that scored clinical factors and created nomograms to predict PFS for first/second generation EGFR‐TKIs, PS was reported as an essential predictor of PFS.^14^ However, in the few real‐world studies on first‐line osimertinib therapy, PS is not listed as an independent predictor of PFS,^21^, ^22^ but the results of this study suggest that PS is an independent predictor of PFS for first‐line osimertinib. Currently, in Japan, gefitinib or erlotinib, first‐generation EGFR‐TKIs, are recommended as first‐line treatment for patients with EGFRm+ advanced NSCLC and a PS of 2, while gefitinib is preferred in patients with a PS of 3–4, based on the evidence of safety and efficacy.^5^, ^28^, ^29^, ^30^ There is insufficient evidence to determine whether osimertinib is more effective than first‐generation EGFR‐TKIs in patients with poor PS. However, the FLAURA study reported that osimertinib is less toxic than first‐generation EGFR‐TKIs, and osimertinib has been administered to patients with poor PS in the real‐world. A phase II trial using gefitinib as first‐line therapy in patients with poor PS reported a PS improvement rate of 79% (90% CI: 67–92),^28^ while a prospective study using osimertinib reported a PS improvement rate of 50% (CI: unknown).^31^ In patients with positive T790M mutation after disease progression, using osimertinib as a second‐line therapy after treatment with first‐generation EGFR‐TKIs is reported to have a potential survival advantage over using osimertinib as first‐line therapy.^32^ Based on these findings, the use of gefitinib, which is expected to improve PS in the first‐line setting, may be preferred for patients with poor PS, followed by the use of osimertinib when the T790M mutation becomes positive after PD onset.

This study found no significant difference in PFS by PD‐L1 TPS. Previous studies have reported PD‐L1 TPS <1% in 42.4%–62.7%, 1%–49% in 25.55%–37.6%, and ≥ 50% in 9.4%–21.2% of lung adenocarcinoma patients.^15^, ^23^, ^33^ PD‐L1 TPS in this study was 49.2% for <1%, 29.5% for 1%–49%, and 21.3% for ≥50%, similar to previously reported results. The association between PD‐L1 TPS and therapeutic efficacy of first/second generation EGFR‐TKIs have been reported in many studies, with patients with PD‐L1 TPS ≥50% reported to have lower objective response rate, shorter PFS, and more de novo resistance compared to those with TPS < 50%.^15^, ^16^, ^17^, ^18^ There are few reports on the association between PD‐L1 TPS and treatment response to osimertinib, but as with first/second generation EGFR‐TKIs, patients with PD‐L1 TPS ≥50% have been reported to have a shorter PFS compared to those with TPS < 50%.^21^, ^23^ In a subset analysis of the FLAURA trial, median PFS was 18.9 months (95% CI: 12.4‐noncalculable) for PD‐L1 TPS <1% and 18.4 months (95% CI: 10.9‐noncalculable) for PD‐L1 TPS ≥1%, suggesting that PFS is not affected by PD‐L1 TPS.^20^ However, only 3.6% of the FLAURA study participants had PD‐L1 TPS ≥50%, which deviates from the real‐world data. It has been reported that patients with PD‐L1 TPS ≥50% had higher baseline blood systemic inflammatory response markers and poorer PS. The FLAURA study excluded patients with PS ≥2, which may have resulted in fewer patients with PD‐L1 TPS ≥50% and, therefore, no difference in median PFS between patients with PD‐L1 TPS <1% and ≥1%. The present study also did not find a significant difference in PFS by PD‐L1 TPS; however, these findings must be interpreted with caution because this was a retrospective study with a small number of patients, and further studies are needed to determine whether PD‐L1 TPS is indeed an independent predictor of PFS in primary osimertinib therapy.

In this study, age was not an independent predictor of PFS. A retrospective study of first‐line osimertinib in patients with EGFRm+ advanced NSCLC aged 75 years or older showed favorable results with a median PFS of 19.4 months (95% CI: 15.9–23.9).^26^ Based on these findings, osimertinib should be considered as a first‐line therapy in patients with EGFRm+ advanced NSCLC, regardless of age.

Liver metastases has been reported as an independent factor negatively affecting PFS for first‐/second‐generation EGFR‐TKIs and osimertinib.^14^, ^21^ Patients with liver metastases tend to have distant metastases in other sites, and the greater the number of metastatic sites, the worse the survival rate, and thus the less likely they are to benefit from EGFR‐TKIs.^11^ In this study, five of the six patients with liver metastasis also had distant metastasis other than liver (pleura: 1, bone: 2, bone and lung: 2). Although liver metastasis was not an independent predictor of PFS in this study, the number of patients with positive liver metastasis was only six (9.9%), and future large‐scale studies are needed to better understand this association.

This study's pattern of adverse events was similar to that of the FLAURA study.^8^ Osimertinib has a limited effect on wild‐type EGFR and tends to have fewer adverse events such as rash and diarrhea than first‐/second‐generation EGFR‐TKIs.^6^, ^8^ In this study, rash, paronychia, and diarrhea were more common, but the incidence of adverse events of grade ≥3 was low (rash 3.3%, no paronychia or diarrhea). The incidence of all grades of interstitial lung disease was 6.6% and of grade 3 was 4.9%, similar to previous reports.^19^

There are several limitations to this study. First, it is a single‐center study, and the sample size is small. In this study, only PS emerged as an independent predictor of PFS, and PD‐L1 TPS or liver metastases did not; these findings differ from the results of previous studies and require further large‐scale investigations. Second, PS was assessed by the treating physician, but subjective bias may not have been eliminated. Third, we could not compare the efficacy of osimertinib with that of first/second‐generation EGFR‐TKIs, because we do not have statistical data on the use of first/second‐generation EGFR‐TKIs in first‐line treatment of EGFRm+ advanced NSCLC patients in our hospital. Fourth, there were no patients with a PS of 4 in our study cohort; therefore, the clinical benefit of osimertinib for that population is unknown.

In conclusion, our study demonstrated in real‐world settings that patients with EGFRm+ advanced NSCLC could have good PFS when osimertinib is used as first‐line therapy. In particular, poor PFS was demonstrated in patients with poor PS. More extensive clinical studies are needed to confirm this finding.

## CONFLICT OF INTEREST

The authors have no conflict of interest to declare.
